# Identification of Long Non-Coding RNA MIR4435-2HG as a Prognostic Biomarker in Bladder Cancer

**DOI:** 10.3390/genes13081462

**Published:** 2022-08-17

**Authors:** Zhiquan Hu, Siquan Ma, Yi Sun, Gongwei Long, Ke Chen

**Affiliations:** 1 Department of Urology, Tongji Hospital, Tongji Medical College, Huazhong University of Science and Technology, Wuhan 430030, China; 2 Hubei Institute of Urology, Tongji Hospital, Tongji Medical College, Huazhong University of Science and Technology, Wuhan 430030, China; 3 Department of Urology, National Cancer Center/National Clinical Research Center for Cancer/Cancer Hospital & Shenzhen Hospital, Chinese Academy of Medical Sciences and Peking Union Medical College, Shenzhen 518116, China

**Keywords:** bladder cancer, lncRNA, prognosis, BCG, biomarkers

## Abstract

The abnormal expression of long non-coding RNAs(lncRNAs) is closely related to the prognosis of patients. This finding may indicate a new target for the treatment of malignant tumors. Non-muscle invasive bladder cancer (NMIBC) is the most common subtype of bladder cancer, and BCG intravesical therapy is the first-line treatment for NMIBC, but about half of NMIBC patients relapse within two years after BCG treatment. Therefore, it is necessary to screen out lncRNAs related to the prognosis and treatment of BGC-resistant patients. Here, we performed differential expression analysis of lncRNAs in the Cancer Genome Atlas (TCGA) and Gene Expression Omnibus (GEO) datasets, and screened *MIR4435-2HG* as the only BCG-response-related lncRNA. Then, the prognosis value of *MIR4435-2HG* was validated in several publicly available cohorts, and confirmed its prognostic value in bladder cancer of different stages. In addition, we also analyzed its genetic alterations, clinical relevance, function enrichment, and association with other biomarkers. Further validation of the expression of *MIR4435-2HG* might be helpful to instruct NMIBC prognosis and treatment.

## 1. Introduction

Bladder cancer is a common malignancy and is the fourth most common cancer in men [1]. With the improvement in diagnostics and technology, the morbidity of bladder cancer has gradually increased in recent years [2]. Although the management of bladder cancer has made great progress in the past two decades, there is a significant rate of recurrence [3].

Bladder cancer is divided into two categories: non-muscle invasive bladder cancer (NMIBC) and muscle invasive bladder cancer (MIBC), according to the depth and level of the invasion of bladder tumor [4]. Although the surgical treatment of NMIBC has a good effect, the one year recurrence rate can reach 15 to 70% [5]. In order to reduce the recurrence of intermediate- and high- risk NMIBC patients, the EAU guidelines recommended that postoperative intravesical instillation therapy should be maintained for more than one year [6]. BCG is the first-line treatment for NMIBC, in patients with intermediate- and high-risk of NMIBC, the effect is more pronounced than that of chemotherapeutic drug infusion, but about half of NMIBC patients still relapse within two years after BCG treatment [7,8]. Additionally, the BCG treatment changed the pattern of patients’ prognosis. The existing NMIBC scale, EORTC and EAU prognostic factor risk groups overestimated the risk of progression in patients treated with BCG, and the EORTC tables overestimated the risk of recurrence in patients treated with BCG [9,10]. The poor prognosis of NMIBC is related to trained immunity, while lncRNA can regulate trained immunity [11,12]. Therefore, lncRNAs could potentially assist the prognosis prediction in BCG-treated patients.

Most RNA in human cells are non-coding RNA (ncRNA), which is classified into micro RNAs(miRNAs), circular RNAs (circRNAs), long non-coding RNAs (lncRNAs), nucleolar RNA (snRNA), and other types of ncRNAs [13,14]. lncRNAs are RNA transcripts with a length of more than 200 nucleotides and play an important role in the biological procession of disease [15]. The abnormal expression of lncRNA is closely associated with prognosis and therapy targets of malignancies [16,17].

The identification of lncRNAs as new biomarkers and prognosis for bladder cancer is promising. In the present study, we used TCGA transcriptome data and GSE176178 data, aiming to identify differentially expressed lncRNA with predictive value for BCG durable NMIBC patients. We screened an only overlap lncRNA which is named *MIR4435-2HG* from the above-mentioned databases. Therefore, *MIR4435-2HG* is likely to be associated with the prognosis of patients with NMIBC treated with BCG. Then, we validated the prognosis value of *MIR4435-2HG* in multiple publicly available datasets. In addition, we also analyze its genetic alterations, clinical relevance, function enrichment, and association with other biomarkers. Accordingly, we proposed to assess *MIR4435-2HG* expression and clinical prognosis of BCG durable NMIBC patients in these databases.

## 2. Materials and Methods

### 2.1. Data Source and Preprocessing

The FPKM-normalized transcriptome data of the Cancer Genome Atlas (TCGA) and the relevant clinical information were downloaded from the UCSC Xena (https://xenabrowser.net/datapages (accessed on 28 November 2021)) [18]. The lncRNAs’ expressions are selected. The count RNA-seq expression data of GSE176178 and FPKM-normalized RNA-seq of GSE154261 were retrieved from the Gene Expression Omnibus (GEO). The transcriptional expression profiling by array in GSE13507 was downloaded from GEO and normalized with the “limma” package [19]. The normalized expression profile and clinical information of the UROMOL cohort were downloaded from the supplementary data of the UROMOL project publication [20]. Information about the above datasets is in Table S1.

### 2.2. Differently Expressed Gene (DEG) Analysis

The DEG of normal and tumor in the TCGA-BLCA cohort were computed using the “limma” package [19]. The count data of GSE176178 were used as input for the “DESeq2” package to identify DEGs between BCG durable and non-durable patients [21]. In GSE176178, BCG non-durable was defined as patients with recurrence of BCa (any stage or grade) within 2 years, and BCG durable had no recurrences detected during follow-up with a disease-free interval of at least 2 years [22]. The threshold for the adjusted *p*-value was set as <0.05, and >0.5 for log2 fold change (logFC).

### 2.3. Survival Analysis

The expression of *MIR4435-2HG* and oncologic outcomes were integrated to assess the prognostic value of *MIR4435-2HG* expression. The oncologic outcomes included overall survival (OS), progression-free survival (PFS), and recurrence-free survival (RFS) if available. The optimal cut-off values of *MIR4435-2HG* expression were calculated by the “surv_cutpoint” function of “survminer” package and the minimal proportion of observations per group was set as 0.2. The expression level was divided into high and low levels according to cut-off values. The Kaplan–Meier survival analysis with log-rank test was applied to estimate the effect of *MIR4435-2HG* level (high vs. low) on patient outcomes. Univariate and multivariate analyses with the Cox proportional hazards model were used to identify independent predictors of BCa outcomes.

### 2.4. Function Enrichment

We utilized cBioPortal to find the *MIR4435-2HG* co-expressed genes in the TCGA-BLCA cohort [23]. Co-expressed genes with a Spearman’s r > 0.6 were selected to perform function enrichment. The clusterProfiler was used to perform gene ontology (GO) and Kyoto encyclopedia of genes and genomes (KEGG) enrichment analysis [24].

### 2.5. Association with Other Biomarkers

The immune infiltrates of the TCGA-BLCA cohort by quanTIseq algorithms were downloaded from the TIMER 2.0 database [25,26]. The homologous recombination deficiency (HRD) score, DNA methylation-based stemness scores (DNAss), and RNA-based stemness scores (RNAss) of the TCGA Pan-Cancer cohort were downloaded from UCSC Xena. The MAF file of TCGA-BLCA was downloaded and the tumor mutational burden (TMB) was calculated with Maftools [27]. The microsatellite instability (MSI) assessed by MANTIS was downloaded from Bonneville’s publication [28]. The associations between *MIR4435-2HG* expression and these biomarkers were evaluated using Spearman’s r.

### 2.6. Statistics Analysis and Codes

All analyses were conducted by R software (version 4.1.1), and the packages were mentioned above. All codes are available on request. Continuous data were compared using the Wilcoxon rank-sum test. Statistical significance was set at *p* < 0.05.

## 3. Results

### 3.1. Identification of Differentially Expressed lncRNA MIR4435-2HG

**Figure 1:**
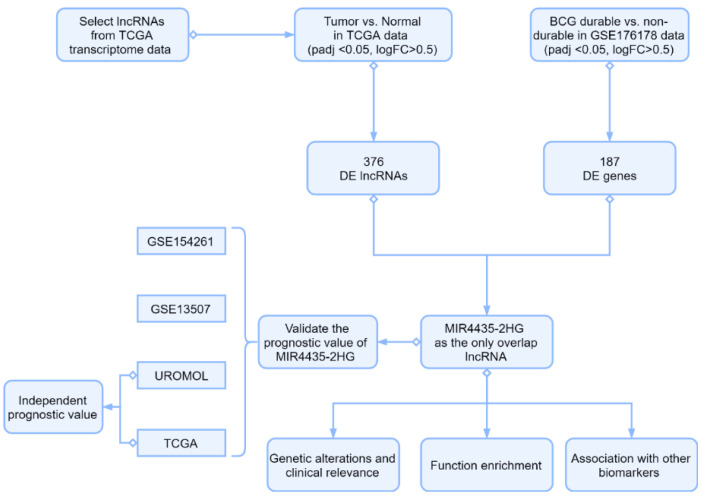
Study workflow. The DEGs of normal and tumor in TCGA-BLCA cohort and BCG non-durable and BCG durable patients in GSE176178 were computed. The *MIR4435-2HG* was screened as the only overlap lncRNA between TCGA and GEO. The prognostic value of *MIR4435-2HG* was validated by multiple cohorts including GSE154261, GSE13507, UROMOL, and TCGA. UROMOL and TCGA were also used to analyze the independent prognostic value of *MIR4435-2HG*. Then, the genetic alterations, clinical relevance, function enrichment, and association with other biomarkers of *MIR4435-2HG* were analyzed.

The flow diagram of this analysis is presented in Figure 1. After screening, 1441 lncRNAs in TCGA dataset were selected for DEG analysis, and 376 differently expressed lncRNAs between normal and BCa tissue were identified. DEG analysis of GSE176178 dataset presented 187 DEGs between BCG durable and non-durable patients (Supplementary Figure S1). Eventually, *MIR4435-2HG* was identified as the only common gene in the two DEG analyses. It is worth noting that the different cohorts consist of different populations: UROMOL is NMIBC, GSE154261 is BCG-treated NMIBC, TCGA is MIBC, and GSE13507 is a mixed cohort of NMIBC and MIBC.

### 3.2. Expressed Level of MIR4435-2HG in Diverse Clinical Groups

**Figure 2:**
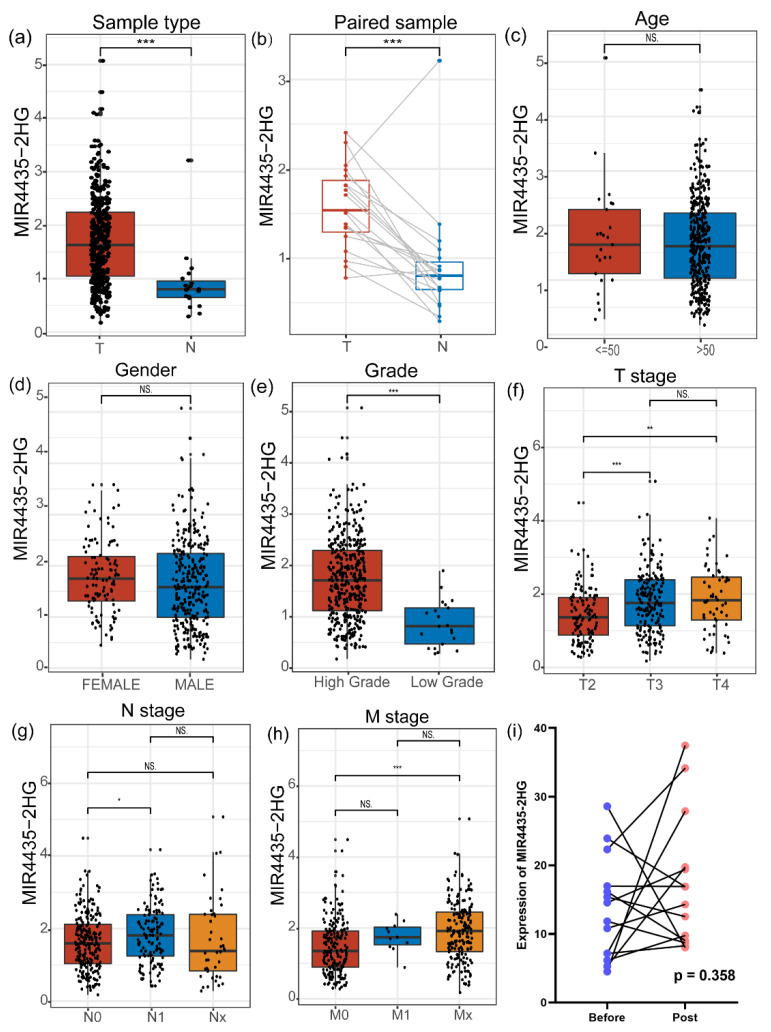
The different expressions of *MIR4435-2HG* based on clinical features. (**a**) *MIR4435-2HG* expression in bladder cancer compared to normal bladder tissues. (**b**) *MIR4435-2HG* levels were compared in paired samples. (**c**) The expression of *MIR4435-2HG* was analyzed in different ages. (**d**) Difference of *MIR4435-2HG* by gender. (**e**) The level of *MIR4435-2HG* according to pathological high grade and low grade. (**f**–**h**) The different expression levels were computed in subgroups according to T stage, N stage and M stage, respectively. (**i**) Paired tissues before and after BCG treatment in 14 patients were analyzed. * *p* < 0.05, ** *p* < 0.01, *** *p* < 0.001, NS (Not significant).

Next, we evaluated the expression of *MIR4435-2HG* from TCGA. Its expression is significantly different between tumor vs. normal group (Figure 2a) and paired sample group (Figure 2b). However, there was no significant difference in the expression of *MIR4435-2HG* between age groups (Figure 2c) and gender groups (Figure 2d). In the grade groups, the level of *MIR4435-2HG* in high grade was markedly higher than in low grade (Figure 2e). Not only that, the TNM stage of bladder cancer was analyzed by Wilcoxon rank-sum test, and the results showed that T stage (Figure 2f), M stage (Figure 2g) and N stage (Figure 2h) were partially significantly associated with the expression of *MIR4435-2HG*. The GSE199471 dataset, in which 14 pairs of bladder tumor tissues retrieved before and after BCG treatment were included, was used to analyze the change of *MIR4435-2HG* expression in bladder tumor tissues. The results suggested that BCG therapy does not directly alter the *MIR4435-2HG* expression in bladder tumor tissues (Figure 2i). Other bypass mechanisms, such as tumor microenvironment, need further explorations to clarify the *MIR4435-2HG*-mediated BCG resistance.

### 3.3. The Prognostic Value of MIR4435-2HG was Confirmed by Survival Analysis

**Figure 3:**
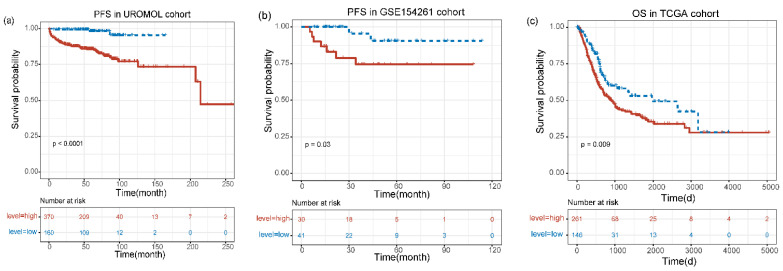
Kaplan–Meier curves for the prognostic value of *MIR4435-2HG*. (**a**) The PFS in UROMOL cohort. (**b**) The PFS in GSE154261 cohort. (**c**) The OS in TCGA cohort. The expression level was divided into high and low levels according to cut-off values.

According to the “surv cutpoint” calculated previously, the expression of *MIR4435-2HG* was divided into high-level and low-level in various datasets that include UROMOL, GSE154261 and TCGA. We divide the UROMOL into low-level groups and high-level groups, and Kaplan–Meier analysis showed that the low-level group had more prominent PFS in the UROMOL dataset than the high-level group (*p* < 0.0001) (Figure 3a). In the GSE154261 cohort, we analyzed the PFS of patients in the low-level group and in the high-level group and the results showed PFS that had discrepancy (*p* = 0.03) (Figure 3b). Similarly, in the TCGA cohort, OS was significant longer in the low-level group than in the high-level group (*p* = 0.009) (Figure 3c). Additionally, the RFS of the patient was analyzed in GSE154261, and there was a marked difference between the low-level group and the high-level group (*p* = 0.0083) (Supplementary Figure S2a). In the GES13507 cohort, the low-level group had a better prognosis than the high-level group (*p* = 0.0049) (Supplementary Figure S2b).

### 3.4. The Genetic Variations, Functional Enrichment and Association with Other Biomarkers of MIR4435-2HG

**Figure 4:**
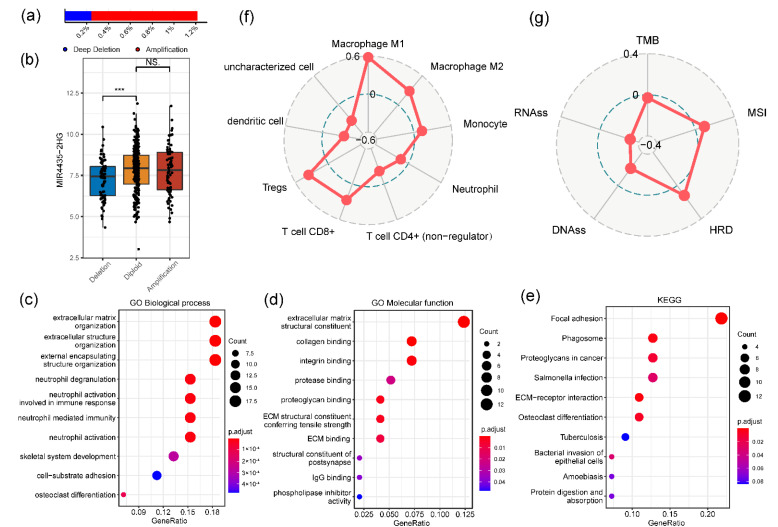
The genetic variations of *MIR4435-2HG* and association with other biomarkers. (**a**,**b**) Genetic variations of *MIR4435-2HG* was assessed by the cBioPortal. *** *p* < 0.001 and NS (Not significant). (**c**–**e**) Gene ontology (GO) and Kyoto encyclopedia of genes genomes (KEGG) enrichment analysis for *MIR4435-2HG*, genes with a Spearman’s r > 0.6 were used as input. (**f**,**g**) Immune infiltrates of TCGA-BLCA and association between *MIR4435-2HG* expression and these biomarkers were evaluated using Spearman’s r.

The cBioPortal was utilized to assess the genetic variations of *MIR4435-2HG* (Figure 4a). In the 411 TCGA-BLCA patients, four high-level copy number amplifications and one deep deletion were detected. Besides, 66 low-level copy number amplifications and 76 shallow deletions were also observed. The *MIR4435-2HG* mRNA expression was reduced in the copy number deletion group, while the copy number amplification did not elevate the *MIR4435-2HG* mRNA level (Figure 4b). To investigate the potential biological processes and functions of *MIR4435-2HG*, the co-expressed genes were used to conduct GO analysis and KEGG pathway analysis. The dot plot of Figure 4c showed that *MIR4435-2HG* was significantly enriched in biological processes (BP) associated with immunity, involving neutrophil degranulation, neutrophil activation involved in immune response and neutrophil mediated immunity. In Figure 4d, the *MIR4435-2HG* was enriched in the extracellular matrix structural constituent related to molecular functions, such as collagen binding and integrin binding. KEGG pathway analysis manifested that *MIR4435-2HG* was enriched in immune-related pathways, which was consistent with the result of anterior GO analysis. The terms included focal adhesion, phagosome and salmonella infection. In addition, the term proteoglycans was related to cancer (Figure 4e). Not only that, the immune infiltrate of the TCGA-BLCA cohort indicated that *MIR4435-2HG* was related to immunity, including Macrophage M1/M2, Monocyte, Tregs and T cell CD8+ (Figure 4f). Other tumor-related biomarkers were also detected (Figure 4g). The aforesaid outcomes demonstrate that *MIR4435-2HG* was highly correlated with cancer and immunity.

### 3.5. The Independent Predictors of BCa Were Identified by Univariate and Multivariate Cox Analyses

We verified independent prognostic values of BCa by Cox proportional hazards model. As shown in Table 1, the *MIR4435-2HG* level and some clinical features were identified as highly correlated with PFS in patients with UROMOL by univariate Cox analysis. We conducted a multivariate Cox analysis of highly correlated predictors, such as *MIR4435-2HG* level (*p* = 0.0002), tumor stage T1 (*p* < 0.0001), tumor grade (*p* < 0.0001), EORTC high risk (*p* < 0.0001), EAU high risk (*p* = 0.0082). The results of multivariate Cox analysis displayed three significantly independent risk factors including high *MIR4435-2HG* level (HR: 9.14, 95% CI: 2.21–37.76, *p* = 0.0022), tumor stage T1 (HR: 2.9, 95% CI: 1.46–5.77, *p* = 0.0024), and high risk according to the EORTC risk table (HR: 7.82, 95% CI: 1.01–60.45, *p* = 0.0488). Similarly, in TCGA, we tested predictors of BCa patients related to OS. The outcome showed these predictors that *MIR4435-2HG* level (Ref: low) (HR: 1.52, 95% CI: 1.08–2.14, *p* = 0.0171), Age (Ref: ≤50 yr) (HR: 2.57, 95% CI: 1.05–6.31, *p* = 0.0389), Stage N1(Ref:N0) (HR: 2.02, 95% CI: 1.46–2.8, *p* < 0.0001) and NX(Ref:N0) (HR: 1.77, 95% CI: 1.08–2.89, *p* = 0.0224) were significant in multivariate Cox analysis (Table 2).

## 4. Discussion

Bladder cancer is a highly prevalent disease with high morbidity and mortality in developed countries [2]. Although great progress has been made in the treatment of bladder cancer, it is still difficult to identify reliable molecular markers for the prognosis of bladder cancer [29]. In recent years, an increasing number of studies have reported that lncRNAs are associated with the pathophysiology and progression of bladder cancer, and they are expected to become new prognostic biomarkers for BCa [30].

BCG immunotherapy is used in the treatment of NMIBC, which is the gold standard for non-recurrent or progressive NMIBC [29]. BCG vaccine induces specific tumor immunity, which may be due to the internalization of the BCG vaccine in the urinary tract epithelium, resulting in the production of cytokines and chemokines, and then recruits a series of immune cells to participate in the immune response [31]. Clinically, the treatment of BCG on NMIBC results in no effect or resistance which are inexplicable [32]. Thus, there is an urgent need to find markers related to BCG response.

The functional annotation of non-coding RNA (ncRNA) is not well-explored, so most of the studies are limited to the prognosis values of protein-coding genes, and there are relatively few studies on ncRNA such as long non-coding RNA [33]. In particular, there are few lncRNA-related studies in NMIBC, and the exploration of the association with BCG treatment response has not been involved [34,35,36]. In the current study, we identified that *MIR4435-2HG* expression may serve as a valuable predictor for BCG durable in patients with NMIBC.

The lncRNA *MIR4435-2HG*, also known as *LINC00978*, is located on human chromosome 2q13. It plays an oncogenic role that is implicated in various tumors [37]. For example, the *MIR4435-2HG* was highly expressed in liver cancer tissue and hepatoma cell lines, and was positively associated with progression and poor prognosis of liver cancer patients [38]. Previous studies have shown that *MIR4435-2HG* promotes the proliferation and invasion of non-small cell lung cancer by inhibiting *miR-6754-5p* expression [39]. Additionally, *MIR4435-2HG* knockdown can inhibit the proliferation, invasion and migration of prostate cancer PC-3 cells by inhibiting FAK/AKT/β-catenin signaling pathway [40]. Moreover, Chen et al. confirmed through experiments that the knockdown of *MIR4435-2HG* can lead to the inactivation of Wnt/β-catenin signaling pathway, further increased cell apoptosis and decreased cell proliferation, migration and invasion [41]. Wang et al. showed that knocked down *MIR4435-2HG* inhibited the cell proliferation, colony formation, and migration of bladder cancer cells. They also demonstrated that *MIR4435-2HG* can sponge *miR-4428* to promote cancer progression; it is expected to become a potential therapeutic target for bladder cancer [42].

In this study, *MIR4435-2HG*, an lncRNA marker, was screened out through the exploration of public data mining. *MIR4435-2HG* showed a certain prognostic value in the cohort of patients with NMIBC and MIBC, and it was also related to the response to BCG perfusion therapy in patients with NMIBC. It can be used to evaluate the clinical prognostic significance of patients with BCa and can be used as a molecular marker target for the treatment of drug resistant patients with BCG. The main drawback of this study is that all the analyses were based on publicly available datasets. However, through the joint verification of *MIR4435-2HG* by multiple cohorts, the prognosis with a significant stratification effect is obtained, and the result is robust enough. In addition, Wang et al. have experimentally verified the function of *MIR4435-2HG* in bladder cancer cells, which supported the important role of *MIR4435-2HG*. However, the downstream targets and related pathways of *miR-4428* still need to be further studied. At the same time, whether *MIR4435-2HG* has an effect on the tumor immune microenvironment and its specific mechanism also needs to be explored.

## 5. Conclusions

In conclusion, we analyzed the public data of multiple bladder cancer cohorts and found that *MIR4435-2HG* has a significant prognostic value. Our findings provide a new insight for the prediction of bladder cancer, not only to provide auxiliary indicators for clinical application to judge the prognosis and to formulate management strategies, but also to provide new directions for experimental research.

## Figures and Tables

**Table 1:** Univariate and multivariate Cox regression of progression-free survival in UROMOL.

Variable	Univariate Analysis		Multivariate Analysis	
	HR (95% CI)	*p*-Value	HR (95% CI)	*p*-Value
*MIR4435-2HG* level (Ref: Low)	9.08 (2.85–28.97)	**0.0002**	9.14 (2.21–37.76)	**0.0022**
Age (Ref: ≤50 yr)	3.11 (0.74–13.08)	0.1217		
Gender (Ref: Female)	1.4 (0.74–2.62)	0.2984		
Tumor stage T1 (Ref: Ta-CIS)	4.59 (2.8–7.53)	**<0.0001**	2.9 (1.46–5.77)	**0.0024**
Tumor grade (Ref: Low)	3.36 (2.01–5.63)	**<0.0001**		
Tumor Size (Ref: <3 cm)	1.46 (0.79–2.69)	0.2242		
EORTC risk High (Ref: Low)	4.42 (2.59–7.55)	**<0.0001**	7.82 (1.01–60.45)	**0.0488**
EAU risk				
Intermediate (Ref: Low)	2.74 (0.62–12.22)	0.1852		
High (Ref: Low)	6.74 (1.64–27.78)	**0.0082**		

*p*-value < 0.05 was considered statistically significant and highlighted in bold.

**Table 2:** Univariate and multivariate Cox regression of overall survival in TCGA.

Variable		Univariate Analysis		Multivariate Analysis	
		HR (95% CI)	*p*-Value	HR (95%CI)	*p*-Value
*MIR4435-2HG* level (Ref: Low)		1.56 (1.11–2.18)	**0.0096**	1.52 (1.08–2.14)	**0.0171**
Age (Ref: ≤50 yr)		2.67 (1.1–6.49)	**0.0307**	2.57 (1.05–6.31)	**0.0389**
Gender (Ref: Female)		0.87 (0.63–1.21)	0.4174		
Stage M	M1 (Ref: M0)	3.2 (1.54–6.65)	**0.0019**		
	Mx (Ref: M0)	1.44 (1.06–1.95)	**0.0184**		
Stage N	N1 (Ref: N0)	2.29 (1.67–3.14)	**<0.0001**	2.02 (1.46–2.8)	**<0.0001**
	NX (Ref: N0)	1.71 (1.05–2.78)	**0.0302**	1.77 (1.08–2.89)	**0.0224**
Stage T	T3 (Ref: T2)	1.2 (0.83–1.74)	0.3417		
	T4 (Ref: T2)	1.05 (0.65–1.68)	0.8423		
Pathologic grade (Ref: Low)		2.9 (0.72–11.72)	0.1353		

*p*-value < 0.05 was considered statistically significant and highlighted in bold.

## Data Availability

Not applicable.

## Supplementary Materials

The following supporting information can be downloaded at: https://www.mdpi.com/article/10.3390/genes13081462/s1, Figure S1: DEG analysis is presented by volcanic maps. (a) lncRNAs in TCGA dataset were screened between normal and BCa tissue (Red indicates an upregulation, Blue is the opposite). (b) DEG analysis of GSE176178 according to BCG durable and non-durable patients (Red indicates an upregulation, Blue is the opposite); Figure S2: The Kaplan–Meier survival analysis with log-rank test was applied to estimate the effect of MIR4435-2HG level (high vs. low) on patient outcomes. (a) The recurrence-free survival (RFS) in GSE154261 cohort. (b) The overall survival (OS) in GSE13507 cohort; Table S1: Information about the dataset.

## References

[1] Lenis A.T., Lec P.M., Chamie K., Mshs M.D. (2020). Bladder Cancer: A Review. Jama.

[2] Sanli O., Dobruch J., Knowles M.A., Burger M., Alemozaffar M., Nielsen M.E., Lotan Y. (2017). Bladder cancer. Nat. Rev. Dis. Prim..

[3] Grossman H.B., Natale R.B., Tangen C.M., Speights V.O., Vogelzang N.J., Trump D.L., de Vere White R.W., Sarosdy M.F., Wood D.P., Raghavan D. (2003). Neoadjuvant chemotherapy plus cystectomy compared with cystectomy alone for locally advanced bladder cancer. N. Engl. J. Med..

[4] Cumberbatch M.G.K., Jubber I., Black P.C., Esperto F., Figueroa J.D., Kamat A.M., Kiemeney L., Lotan Y., Pang K., Silverman D.T. (2018). Epidemiology of Bladder Cancer: A Systematic Review and Contemporary Update of Risk Factors in 2018. Eur. Urol..

[5] Sylvester R.J., van der Meijden A.P., Oosterlinck W., Witjes J.A., Bouffioux C., Denis L., Newling D.W., Kurth K. (2006). Predicting recurrence and progression in individual patients with stage Ta T1 bladder cancer using EORTC risk tables: A combined analysis of 2596 patients from seven EORTC trials. Eur. Urol..

[6] Babjuk M., Burger M., Compérat E.M., Gontero P., Mostafid A.H., Palou J., van Rhijn B.W., Rouprêt M., Shariat S.F., Sylvester R. (2019). European Association of Urology Guidelines on Non-muscle-invasive Bladder Cancer (TaT1 and Carcinoma in Situ)-2019 Update. Eur. Urol..

[7] Kates M., Matoso A., Choi W., Baras A.S., Daniels M.J., Lombardo K., Brant A., Mikkilineni N., McConkey D.J., Kamat A.M. (2020). Adaptive Immune Resistance to Intravesical BCG in Non-Muscle Invasive Bladder Cancer: Implications for Prospective BCG-Unresponsive Trials. Clin. Cancer Res..

[8] Oddens J., Brausi M., Sylvester R., Bono A., van de Beek C., van Andel G., Gontero P., Hoeltl W., Turkeri L., Marreaud S. (2013). Final results of an EORTC-GU cancers group randomized study of maintenance bacillus Calmette-Guérin in intermediate- and high-risk Ta, T1 papillary carcinoma of the urinary bladder: One-third dose versus full dose and 1 year versus 3 years of maintenance. Eur. Urol..

[9] Fernandez-Gomez J., Madero R., Solsona E., Unda M., Martinez-Piñeiro L., Ojea A., Portillo J., Montesinos M., Gonzalez M., Pertusa C. (2011). The EORTC tables overestimate the risk of recurrence and progression in patients with non-muscle-invasive bladder cancer treated with bacillus Calmette-Guérin: External validation of the EORTC risk tables. Eur. Urol..

[10] Lobo N., Hensley P.J., Bree K.K., Nogueras-Gonzalez G.M., Navai N., Dinney C.P., Sylvester R.J., Kamat A.M. (2022). Updated European Association of Urology (EAU) Prognostic Factor Risk Groups Overestimate the Risk of Progression in Patients with Non-muscle-invasive Bladder Cancer Treated with Bacillus Calmette-Guérin. Eur. Urol. Oncol..

[11] Acevedo O.A., Berrios R.V., Rodríguez-Guilarte L., Lillo-Dapremont B., Kalergis A.M. (2021). Molecular and Cellular Mechanisms Modulating Trained Immunity by Various Cell Types in Response to Pathogen Encounter. Front. Immunol..

[12] Fanucchi S., Mhlanga M.M. (2019). Lnc-ing Trained Immunity to Chromatin Architecture. Front. Cell Dev. Biol..

[13] Esteller M. (2011). Non-coding RNAs in human disease. Nat. Rev. Genet..

[14] Goodall G.J., Wickramasinghe V.O. (2021). RNA in cancer. Nat. Rev. Cancer.

[15] Cech T.R., Steitz J.A. (2014). The noncoding RNA revolution-trashing old rules to forge new ones. Cell.

[16] Anastasiadou E., Jacob L.S., Slack F.J. (2018). Non-coding RNA networks in cancer. Nat. Rev. Cancer.

[17] Matsui M., Corey D.R. (2017). Non-coding RNAs as drug targets. Nat. Rev. Drug Discov..

[18] Goldman M.J., Craft B., Hastie M., Repečka K., McDade F., Kamath A., Banerjee A., Luo Y., Rogers D., Brooks A.N. (2020). Visualizing and interpreting cancer genomics data via the Xena platform. Nat. Biotechnol..

[19] Ritchie M.E., Phipson B., Wu D., Hu Y., Law C.W., Shi W., Smyth G.K. (2015). Limma powers differential expression analyses for RNA-sequencing and microarray studies. Nucleic Acids Res..

[20] Lindskrog S.V., Prip F., Lamy P., Taber A., Groeneveld C.S., Birkenkamp-Demtröder K., Jensen J.B., Strandgaard T., Nordentoft I., Christensen E. (2021). An integrated multi-omics analysis identifies prognostic molecular subtypes of non-muscle-invasive bladder cancer. Nat. Commun..

[21] Love M.I., Huber W., Anders S. (2014). Moderated estimation of fold change and dispersion for RNA-seq data with DESeq2. Genome Biol..

[22] Sanders J.A., Frasier C., Matulay J.T., Steuerwald N.M., Zhu J., Grigg C.M., Kearns J.T., Riggs S.B., Gaston K.E., Brouwer C.R. (2021). Genomic analysis of response to bacillus Calmette-Guérin (BCG) treatment in high-grade stage 1 bladder cancer patients. Transl. Androl. Urol..

[23] Gao J., Aksoy B.A., Dogrusoz U., Dresdner G., Gross B., Sumer S.O., Sun Y., Jacobsen A., Sinha R., Larsson E. (2013). Integrative analysis of complex cancer genomics and clinical profiles using the cBioPortal. Sci. Signal..

[24] Wu T., Hu E., Xu S., Chen M., Guo P., Dai Z., Feng T., Zhou L., Tang W., Zhan L. (2021). clusterProfiler 4.0: A universal enrichment tool for interpreting omics data. Innovation.

[25] Li T., Fu J., Zeng Z., Cohen D., Li J., Chen Q., Li B., Liu X.S. (2020). TIMER2.0 for analysis of tumor-infiltrating immune cells. Nucleic Acids Res..

[26] Finotello F., Mayer C., Plattner C., Laschober G., Rieder D., Hackl H., Krogsdam A., Loncova Z., Posch W., Wilflingseder D. (2019). Molecular and pharmacological modulators of the tumor immune contexture revealed by deconvolution of RNA-seq data. Genome Med..

[27] Mayakonda A., Lin D.C., Assenov Y., Plass C., Koeffler H.P. (2018). Maftools: Efficient and comprehensive analysis of somatic variants in cancer. Genome Res..

[28] Bonneville R., Krook M.A., Kautto E.A., Miya J., Wing M.R., Chen H.Z., Reeser J.W., Yu L., Roychowdhury S. (2017). Landscape of Microsatellite Instability Across 39 Cancer Types. JCO Precis. Oncol..

[29] Pettenati C., Ingersoll M.A. (2018). Mechanisms of BCG immunotherapy and its outlook for bladder cancer. Nat. Rev. Urol..

[30] Li Y., Li G., Guo X., Yao H., Wang G., Li C. (2020). Non-coding RNA in bladder cancer. Cancer Lett..

[31] Larsen E.S., Joensen U.N., Poulsen A.M., Goletti D., Johansen I.S. (2020). Bacillus Calmette-Guérin immunotherapy for bladder cancer: A review of immunological aspects, clinical effects and BCG infections. APMIS.

[32] Han J., Gu X., Li Y., Wu Q. (2020). Mechanisms of BCG in the treatment of bladder cancer-current understanding and the prospect. Biomed. Pharmacother..

[33] Quan J., Pan X., Zhao L., Li Z., Dai K., Yan F., Liu S., Ma H., Lai Y. (2018). LncRNA as a diagnostic and prognostic biomarker in bladder cancer: A systematic review and meta-analysis. OncoTargets Ther..

[34] Avgeris M., Tsilimantou A., Levis P.K., Tokas T., Sideris D.C., Stravodimos K., Ardavanis A., Scorilas A. (2018). Loss of GAS5 tumour suppressor lncRNA: An independent molecular cancer biomarker for short-term relapse and progression in bladder cancer patients. Br. J. Cancer.

[35] Zhan Y., Du L., Wang L., Jiang X., Zhang S., Li J., Yan K., Duan W., Zhao Y., Wang L. (2018). Expression signatures of exosomal long non-coding RNAs in urine serve as novel non-invasive biomarkers for diagnosis and recurrence prediction of bladder cancer. Mol. Cancer.

[36] Zhang S., Du L., Wang L., Jiang X., Zhan Y., Li J., Yan K., Duan W., Zhao Y., Wang L. (2019). Evaluation of serum exosomal LncRNA-based biomarker panel for diagnosis and recurrence prediction of bladder cancer. J. Cell. Mol. Med..

[37] Wang H., Wu M., Lu Y., He K., Cai X., Yu X., Lu J., Teng L. (2019). LncRNA MIR4435-2HG targets desmoplakin and promotes growth and metastasis of gastric cancer by activating Wnt/β-catenin signaling. Aging.

[38] Zhu Y., Li B., Xu G., Han C., Xing G. (2022). lncRNA MIR4435-2HG promotes the progression of liver cancer by upregulating B3GNT5 expression. Mol. Med. Rep..

[39] Li X., Ren Y., Zuo T. (2018). Long noncoding RNA LINC00978 promotes cell proliferation and invasion in non-small cell lung cancer by inhibiting miR-6754-5p. Mol. Med. Rep..

[40] Xing P., Wang Y., Zhang L., Ma C., Lu J. (2021). Knockdown of lncRNA MIR4435-2HG and ST8SIA1 expression inhibits the proliferation, invasion and migration of prostate cancer cells in vitro and in vivo by blocking the activation of the FAK/AKT/β-catenin signaling pathway. Int. J. Mol. Med..

[41] Chen D., Tang P., Wang Y., Wan F., Long J., Zhou J., Zhuang M., Chen X. (2021). Downregulation of long non-coding RNA MR4435-2HG suppresses breast cancer progression via the Wnt/β-catenin signaling pathway. Oncol. Lett..

[42] Wang W., Xu Z., Wang J., Chen R. (2019). LINC00978 promotes bladder cancer cell proliferation, migration and invasion by sponging miR-4288. Mol. Med. Rep..

