# Supplementary material for: Identification of Long Non-Coding RNA MIR4435-2HG as a Prognostic Biomarker in Bladder Cancer
Source: Genes (Basel). 2022 Aug 17;13(8):1462. doi: 10.3390/genes13081462 (PMC9408477; doi:10.3390/genes13081462)
Supplement: Supplementary file 1 [file genes-13-01462-s001.zip › genes-1796191-SI.pdf]

Supplementary Materials：

Figure S1: DEG analysis is presented by volcanic maps 2
Figure S2: The Kaplan–Meier survival analysis with log-rank test was applied to estimate the effect of MIR4435-2HG level (high vs. low) on patient outcomes 3
Table S1 Information about the dataset 3

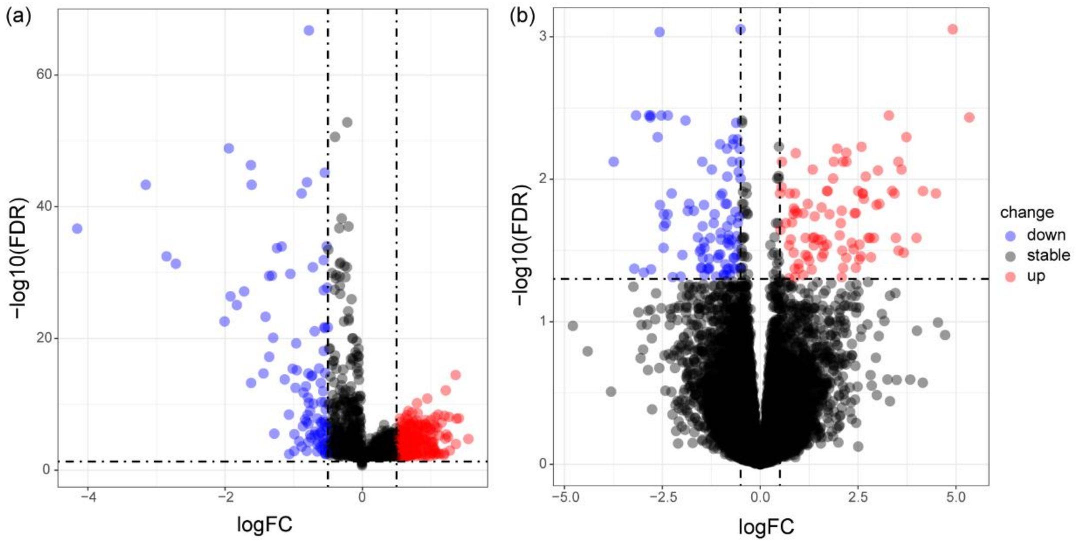

Figure S1: DEG analysis is presented by volcanic maps. (a) lncRNAs in TCGA dataset were screened between normal and BCa tissue (Red indicates an upregulation, Blue is the opposite). (b) DEG analysis of GSE176178 according to BCG durable and non-durable patients (Red indicates an upregulation, Blue is the opposite).

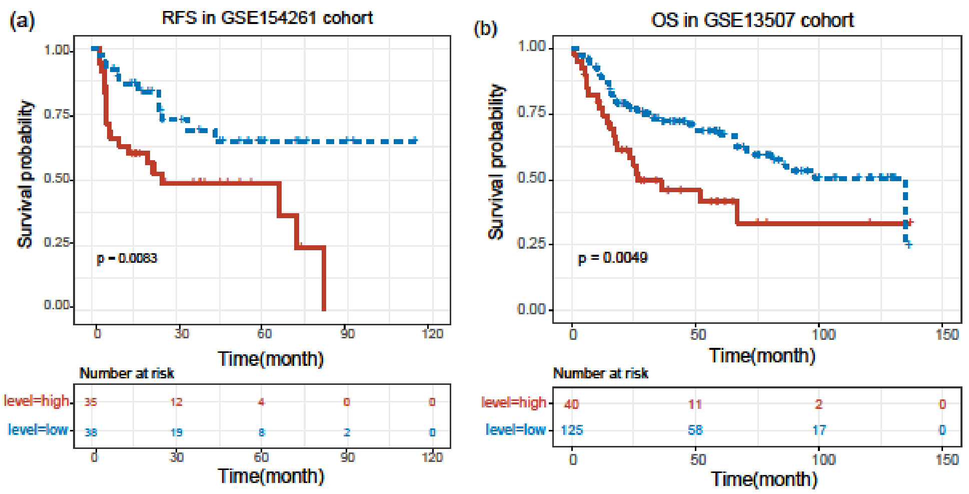

Figure S2: The Kaplan–Meier survival analysis with log-rank test was applied to estimate the effect of MIR4435-2HG level (high vs. low) on patient outcomes. (a) The recurrence-free survival (RFS) in GSE154261 cohort. (b) The overall survival (OS) in GSE13507 cohort.

Table S1 Information about the dataset

Dataset	Quantitative method	Number of samples	Patient information	Research endpoints	PMID
GSE176178	RNA-Seq	40	**HG** T1 NMIBC treated with BCG	BCG response	34430403
GSE154261	RNA-Seq	73	**HG** T1 NMIBC treated with BCG	RFS, PFS	32684305
UROMOL	RNA-Seq	535	NMIBC	RFS, PFS	33863885
TCGA	RNA-Seq	407	MIBC	PFS, OS	29625055
GSE13507	CHIP	165	104 NMIBC, 61 MIBC	OS	20059769

HG: high-grade pathology
